# ^11^C-acetate PET/MRI in bladder cancer staging and treatment response evaluation to neoadjuvant chemotherapy: a prospective multicenter study (ACEBIB trial)

**DOI:** 10.1186/s40644-018-0158-4

**Published:** 2018-08-02

**Authors:** Antti Salminen, Ivan Jambor, Harri Merisaari, Otto Ettala, Johanna Virtanen, Ilmari Koskinen, Erik Veskimae, Jukka Sairanen, Pekka Taimen, Jukka Kemppainen, Heikki Minn, Peter J. Boström

**Affiliations:** 1 Department of Urology, University of Turku and Turku University hospital, Kiinamyllynkatu 4-8, 20520 Turku, Finland; 2 Department of Radiology, University of Turku and Turku University Hospital, Turku, Finland; 3 Department of Urology, University of Helsinki and Helsinki University hospital, Helsinki, Finland; 4 Department of Urology, University of Tampere and Tampere University hospital, Tampere, Finland; 5 Department of Pathology, Institute of Biomedicine, University of Turku and Turku University hospital, Turku, Finland; 6 Department of Clinical Physiology and nuclear imaging, University of Turku and Turku University hospital, Turku, Finland; 7 Department of Oncology and Radiotherapy, University of Turku and Turku University hospital, Turku, Finland; 8 Department of Radiology, Icahn School of Medicine at Mount Sinai, New York, USA

**Keywords:** Bladder cancer, ^11^C-acetate, PET/MRI, Neoadjuvant chemotherapy

## Abstract

**Background:**

To evaluate the accuracy of ^11^C-acetate Positron Emission Tomography/Magnetic Resonance Imaging (PET/MRI) in bladder cancer (BC) staging and monitoring response to neoadjuvant chemotherapy (NAC).

**Methods:**

Eighteen patients were prospectively enrolled. Fifteen treatment naive patients underwent ^11^C-acetate PET/MRI before transurethral resection of bladder tumor (TUR-BT) for primary tumor evaluation. Five patients with muscle invasive BC were imaged after NAC and prior to radical cystectomy (RC) with extended pelvic lymph node dissection (ePLND) for NAC treatment response evaluation. Two patients were part of both cohorts. ^11^C-acetate PET/MRI findings were correlated with histopathology. Accuracy for lymph node detection was evaluated on patient and the ePLND template (10 regions) levels.

**Results:**

The sensitivity, specificity and accuracy of ^11^C-acetate PET/MRI for the detection of muscle invasive BC was 1.00, 0.69 and 0.73 while the area under the receiver operating characteristic curve (95% confidence interval) was 0.85 (0.55–1.0), respectively. All five NAC patients underwent chemotherapy as planned and ^11^C-acetate PET/MRI correctly staged three patients, overstaged one and understaged one patient compared with RC and ePLND findings. A total of 175 lymph node were removed, median of 35 (range, 27–43) per patient in five patients who had RC and ePLND while 12 (7%) harboured metastases. Sensitivity, specificity, accuracy and AUC for N-staging were 0.20, 0.96, 0.80 and 0.58 on the ePLND template (10 regions) level.

**Conclusions:**

^11^C-acetate PET/MRI is feasible for staging of BC although sensitivity for the detection of nodal metastases is low. Monitoring response to NAC shows promise and warrants evaluation in larger studies.

**Trial registration:**

ClinicalTrials.gov Identifier: NCT01918592, registered August 8 2013

## Background

Approximately 77,000 new cases of bladder cancer (BC) are diagnosed annually in USA [1]. In Europe, based on EU science hub estimates, incidence of new BC cases was 131,000 in 2015, but there is large variability between different countries [2]. Staging is based on TNM system [3]. Ta tumors are treated with transurethral resection of bladder tumor (TUR-BT). T1 and carcinoma in situ (Tis) tumors have risk of progression and intravesical immunotherapy with Bacillus-Calmette-Guerin (BCG) instillations is used to obtain local control and organ preservation [4]. Muscle invasive bladder cancer (MIBC) is an aggressive disease and standard treatment is radical cystectomy (RC), accompanied by pelvic lymph node dissection (PLND) [5]. In addition to radical surgery, neoadjuvant chemotherapy (NAC) has been demonstrated to increase overall survival in MIBC and is recommended by consensus guidelines [5, 6].

Staging of BC remains suboptimal with standard imaging modalities such as computed tomography (CT) and anatomical (T2- and T1-weighted imaging) magnetic resonance imaging (MRI) [7, 8]. Compared to contrast enhanced CT, MRI has better soft tissue contrast which may improve local tumor evaluation [9]. Locoregional staging in particular is difficult, since normal-sized lymph nodes may harbour metastases. The use of ultra-small paramagnetic particles of iron oxide (USPIO) has shown some promise in the detection of nodal metastases with MRI [10, 11] but still remains an experimental approach with very limited availability.

Positron emission tomography-CT (PET/CT) imaging has been widely investigated in BC in an attempt to improve nodal staging but with limited success [12–15]. PET/MRI is a novel combination of two imaging modalities which could potentially offer advantages for evaluation of MIBC over PET/CT. Only a limited experience in staging of BC is available with PET/MRI and specifically studies of tracers with low excretion in urine are lacking [16]. Hence, we undertook the current study to evaluate ^11^C-acetate PET/MRI in initial staging and estimation of response to NAC in patients with BC.

## Methods

### Patients

The study was approved by the ethical committee of Hospital District of Southwest Finland and registered at ClinicalTrials.gov (NCT01918592). Candidates for study were screened in cystoscopy units in three participating university hospitals. Eligible patients signed written consent after verbal and written information.

### Imaging protocol

All patients were imaged with an Ingenuity TF PET/MRI scanner (Philips Medical Systems, Cleveland, OH). Details of the physical performance of the system have already been reported [17]. Before imaging, each patient had a Foley catheter inserted holding 10 ml glycine in balloon and the bladder was consequently filled with 100 ml sterile saline. The patients were placed supine on the scanner table. After completed MR-based attenuation correction (see below) the table was rotated for PET scan and a median dose of 713 (range, 654–796) MBq of ^11^C-acetate was injected intravenously and PET acquisition (two 4-min table positions) covering the whole pelvis was immediately started.

MR-based attenuation correction (MRAC) was performed using the vendor-provided method. The anatomical MR acquisition (atMR) for MRAC was performed using Repetition Time/Echo Time (TR/TE) 4.0/2.3 ms and flip angle 10°. Subsequently, MRAC algorithm converts the atMR images by segmentation and classification to attenuation correction maps containing air (0.0 cm^− 1^), lung (0.022 cm^− 1^) and soft tissue (0.096 cm^− 1^). This MRAC map was then used in PET image reconstruction. All quantitative corrections were applied to this reconstruction taking into account detector dead time, radioactivity decay, random, scatter and photon attenuation. PET images were reconstructed in a 144 × 144 matrix with an isotropic voxel size of 4 mm following a fully 3-D maximum-likelihood ordered subsets expectation maximum (LM-OSEM) algorithm with 3 iterations and 33 subsets using TOF technology.

After the PET scan, bladder was emptied and radioactive urine was safely disposed. Bladder was re-filled with 100 ml sterile saline and MRI examination was performed.

T2-weighted imaging was performed using single-shot turbo spin echo sequence with TR/TE 3618/130, field of view (FOV) 300 × 300 mm^2^, acquisition voxel size 1.25 × 1.25 × 3.00 mm^3^, reconstruction voxel size 0.69 × 0.69 × 3.00 mm^3^ in axial (acquisition time 1:30 min), sagittal (acquisition time 1:30 min) and coronal (acquisition time 1:12 min) planes. Diffusion weighted imaging (DWI) covering whole pelvis was performed using a single shot SE-EPI sequence, monopolar diffusion gradient scheme, and the following parameters: TR/TE 3148/45, FOV 300 × 300 cm^2^, acquisition voxel size 2.5 × 2.5 × 4.0 mm^3^, reconstruction voxel size 1.25 × 1.25 × 4.00 mm^3^, b value 0 and 800 s/mm^2^, diffusion gradient timing (Δ) 21.6 ms, diffusion gradient duration (δ) 8.3 ms, diffusion time (Δ-δ/3) 18.8 ms, SENSE [18] factor of 2, partial-Fourier acquisition 0.69, SPAIR fat suppression acquisition time 1:47 min per position (two positions in total). Additional T2-weighted imaging in axial plane with the same slice location as DWI covering whole pelvis was obtained using TR/TE 3148/45, FOV 300 × 300 cm^2^, acquisition voxel size acquisition voxel size 1.25 × 1.25 × 4.00 mm^3^, reconstruction voxel size 0.69 × 0.69 × 4.00 mm^3^, acquisition time 1:05 min per position (two positions in total). Additional B0 and B1 mapping were performed. Multiple additional experimental DWI acquisitions, a spin locking method were acquired but not analyzed in the current study [19]. Detailed importable PET/MRI protocol is provided in the supporting material.

**Fig. 1:**
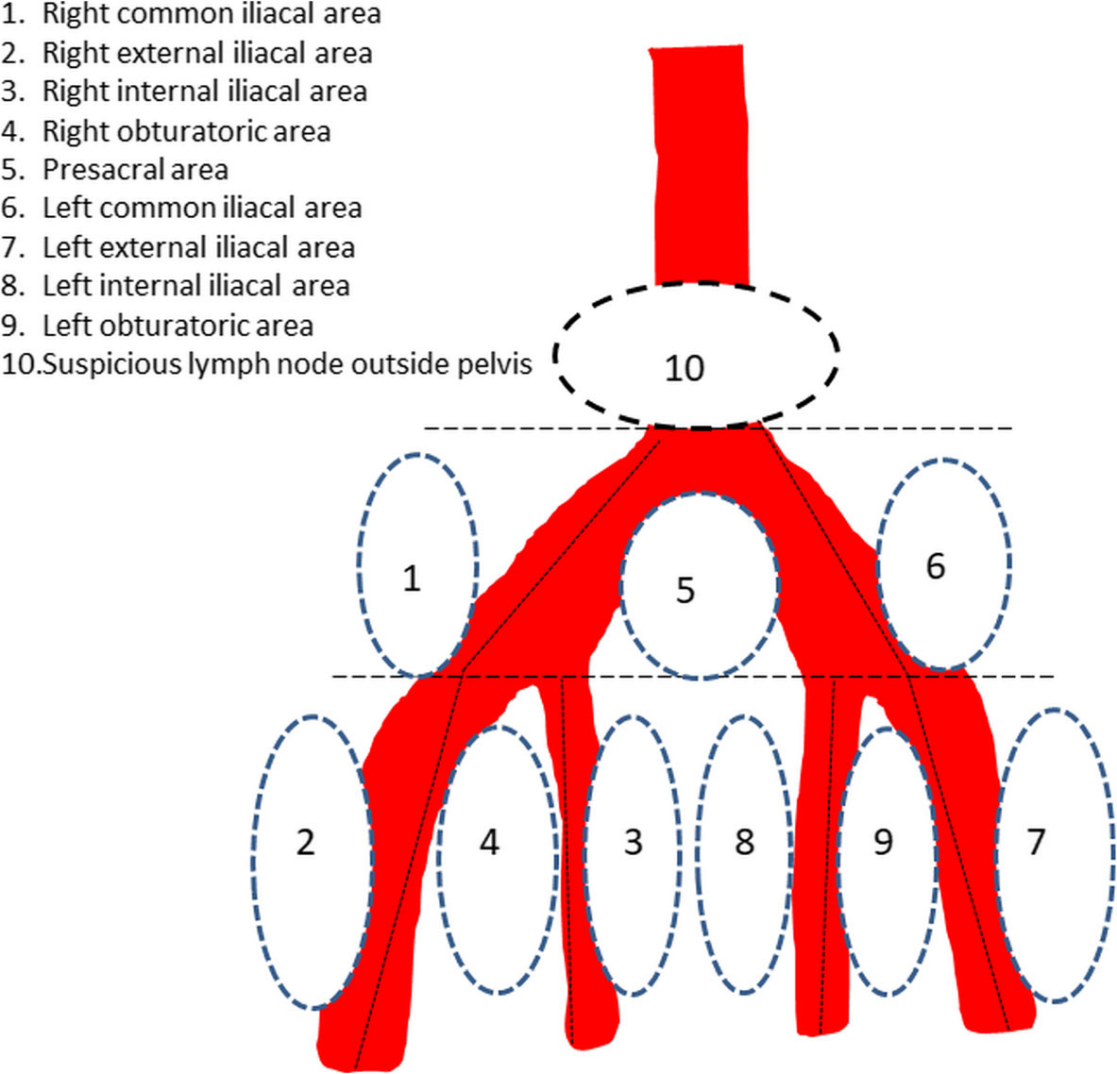
Examined lymph node areas in radical cystectomy

PET/MRI images were evaluated by two radiologists (IJ, JV) in conjunction with an experienced nuclear medicine physician (JK). Primary tumor size, possible muscle invasion and metastatic spread was evaluated [20]. Lymph nodes were evaluated on pre-determined 10 regions (suspicion of metastasis vs. benign) as described in Fig. 1.

### Treatment

**Fig. 2:**
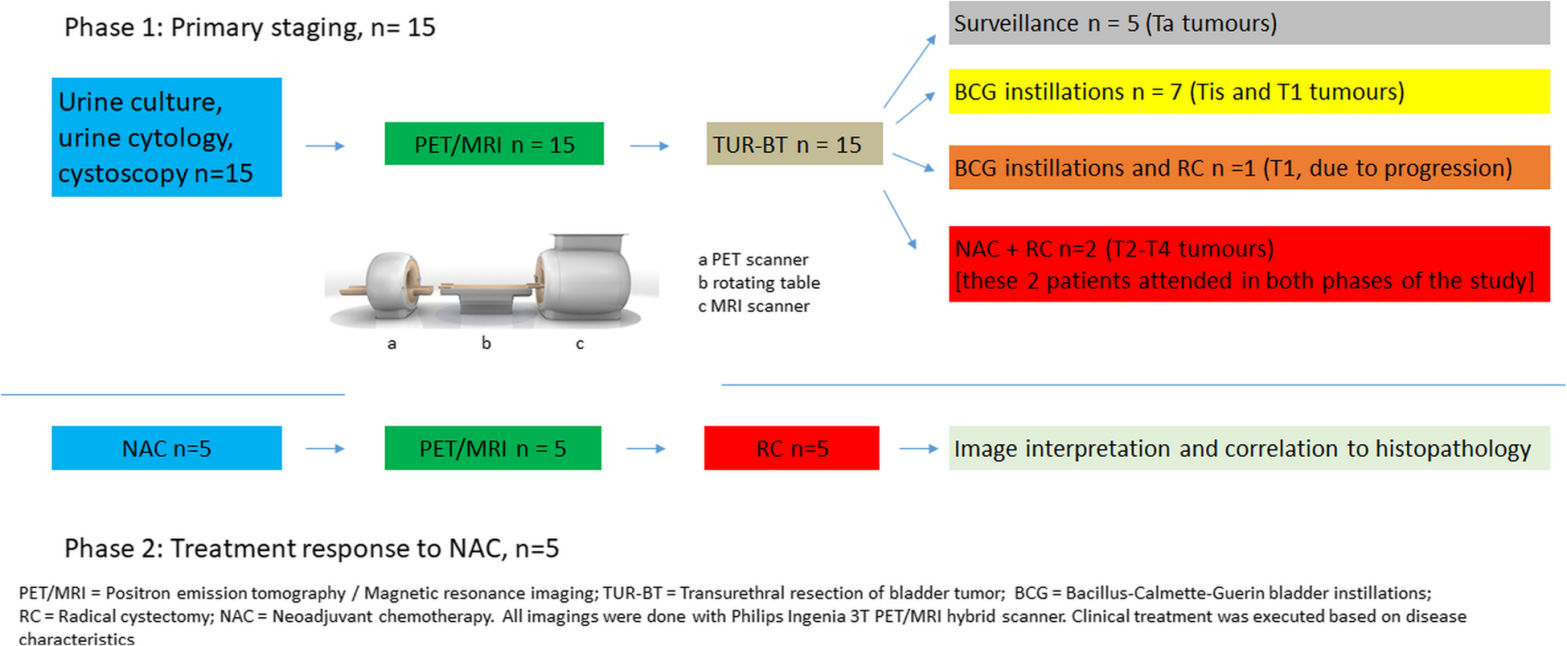
Flow chart of the study protocol. The trial consisted of two phases: in phase 1 accuracy of ^11^C-acetate PET/MRI was evaluated on 15 treatment naive patients before any intervention of primary tumor. In phase 2 treatment response to Neoadjuvant chemotherapy (NAC) was evaluated in 5 patients undergoing ^11^C-acetate PET/MRI after transurethral resection of bladder tumor (TUR-BT) and neoadjuvant chemotherapy. 2 patients participated in both phases of the study. In total, 18 patients were enrolled

A diagnostic TUR-BT followed imaging after a median of 11 (range 1–21) days (Fig. 2) in the primary tumor evaluation of 15 patients. A complete resection was performed in each case and imaging findings did not affect the extent of the procedure. Further treatment after TUR-BT was done according to standard guidelines [5, 21]. In addition to 15 patients who underwent PET/MRI *before* TUR-BT, three MIBC patients were enrolled *after* TUR-BT for NAC treatment response evaluation.

NAC treatment response was evaluated in five patients (Table 1). Two patients in this study phase were part of both phases. Five patients received either 3 or 4 cycles of cisplatin-gemcitabine according to treating hospital policy. Second ^11^C-acetate PET/MRI imaging was performed a median of 21 (range, 11–33) days after completion of NAC. Finally, open RC and extended (E) PLND were performed according to predetermined ten fields described in Fig. 1. Delay from NAC completion to RC was 36 days (range 28-47 days). The selected EPLND template was based on commonly known lymphatic spread pathways [22]. In addition to extent of primary tumour, each predetermined PLND area was separately analysed from the PET/MRI data sets. For the histopathological examination, the RC specimen and the ten PLND areas were sent separately and were analysed by an experienced uropathologist (PT).

**Table 1:** Patients’ characteristics

	Primary tumor	NAC^a^
No of patients in phase of study	15	5
Gender (Male/Female)	13/2	5
Age (years)	67 (55–79)	65 (57–69)
ASA-score	2 (1–3)	2
BMI (kg/m^2^)	27 (23–31)	26 (23–30)
Treatment modality		
TUR-BT and surveillance	5	N/A
TUR-BT and BCG	7	N/A
TUR-BT, NAC, RC and EPLND	2	5
TUR-BT, BCG and RC^b^	1	N/A

Mean values and range are given, when feasible. *ASA-score* The American Society of Anesthesiologists physical status grading system; *BMI* body mass index, *TUR-BT* Transurethral resection of bladder tumor, *BCG* Bacillus-Calmette-Guerin bladder instillations, *RC* radical cystectomy, *EPLND* extended pelvic lymph node dissection, *N/A* not applicable

^a^2 patients participated in both phases of the study

^b^RC due to disease progression

### Statistics

The statistical analysis was performed using Matlab (version r2013a, The MathWorks Inc., Natick, MA). Sensitivity, specificity and accuracy of ^11^C-acetate PET/MRI for BT staging in 15 patients who underwent PET/MRI before TUR-BT was compared with TUR-BT histopathology specimens as ground truth. The classification was performed in binary class: benign + non-muscle invasive (Ta/Tis/T1) vs muscle invasive (T2-T4). In five patients presenting with MIBC who underwent RC and PLND, regional classification (10 regions of interest, in total 50 regions) between benign vs malignant LNs was performed. Additional patient level analysis was performed. Receiver operating characteristic curve analysis was used to evaluate ability of ^11^C-acetate PET/MRI for primary staging and detection of lymph node metastases. Area under the receiver operating characteristic curve (AUC) values were calculated using the trapezoid rule. Ninety-five percent confidence interval for AUC values were calculated from 100,000 bootstrap samples. SUV measurements were compared using Bonferroni multiple comparison test [23].

## Results

In total, 15 patients underwent the primary tumour evaluation and five response evaluation with two patients participating in both groups giving a total of 18 participants. Patient characteristics are presented in Table 1. Despite careful and hygienic catheterization three patients (17%, 3/18) developed urinary tract infection after imaging. Two patients subsequently required hospitalization while one patient received antimicrobial treatment at home. In these three patients underlying bacteriuria could not be ruled out since bacteriuria had not been screened before or at the time of enrolment.

### Primary staging

All primary tumors were urothelial cancer and demonstrated positive uptake of ^11^C-acetate. The median maximum standardized uptake value (SUVmax) was 2.9 (range, 1.3–4.7). There was no difference between SUVmax of MIBC versus superficial (clinical stage Tis-T1) BC.

**Fig. 3:**
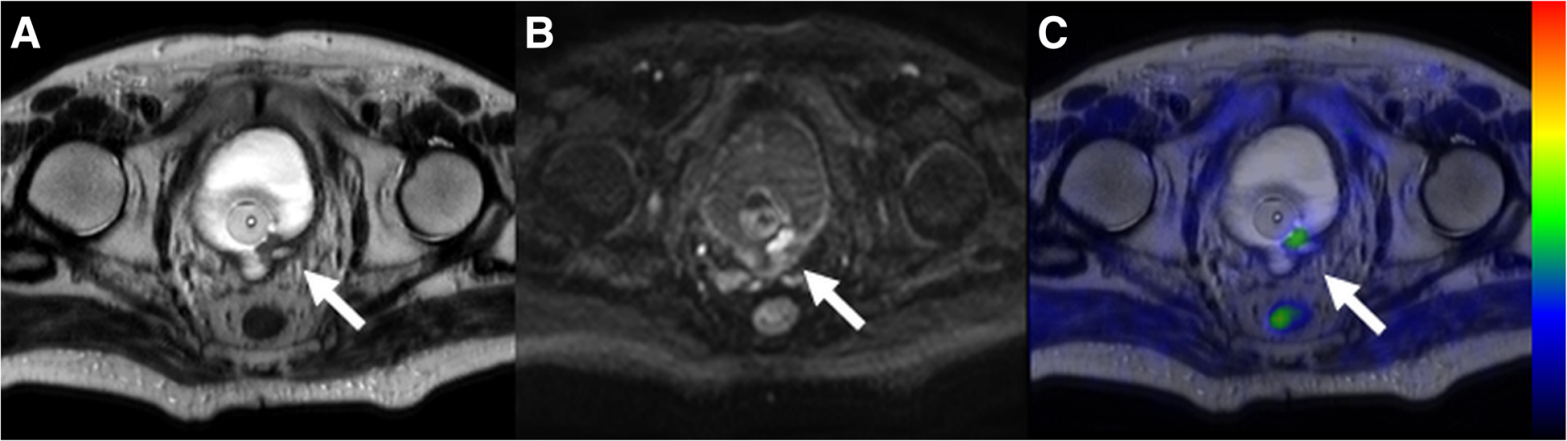
Pre-transurethral resection ^11^C-acetate PET/MRI (**a**, **b**, **c**) in a 75-year old male patient (number 3 in primary imaging) showing a heterogenous lobulated mass on the left side of mid-line **without** extension (white arrow) to perivesical fat on T2-weighted image (**a**) or an area of increased diffusion signal restriction (white arrow) extending beyond the bladder wall (**b** - b value 800 s/mm^2^ trace diffusion weighted image). The lesion had increased ^11^C-acetate uptake (**c** - PET fused with T2-weighted image, SUV is scaled from 0.0 to 3.2). The imaging findings were suggestive of T1 stage. The transurethral resection of bladder tumor specimen revealed stage T1, thus the findings of ^11^C-acetate PET/MRI correctly staged the tumor

**Fig. 4:**
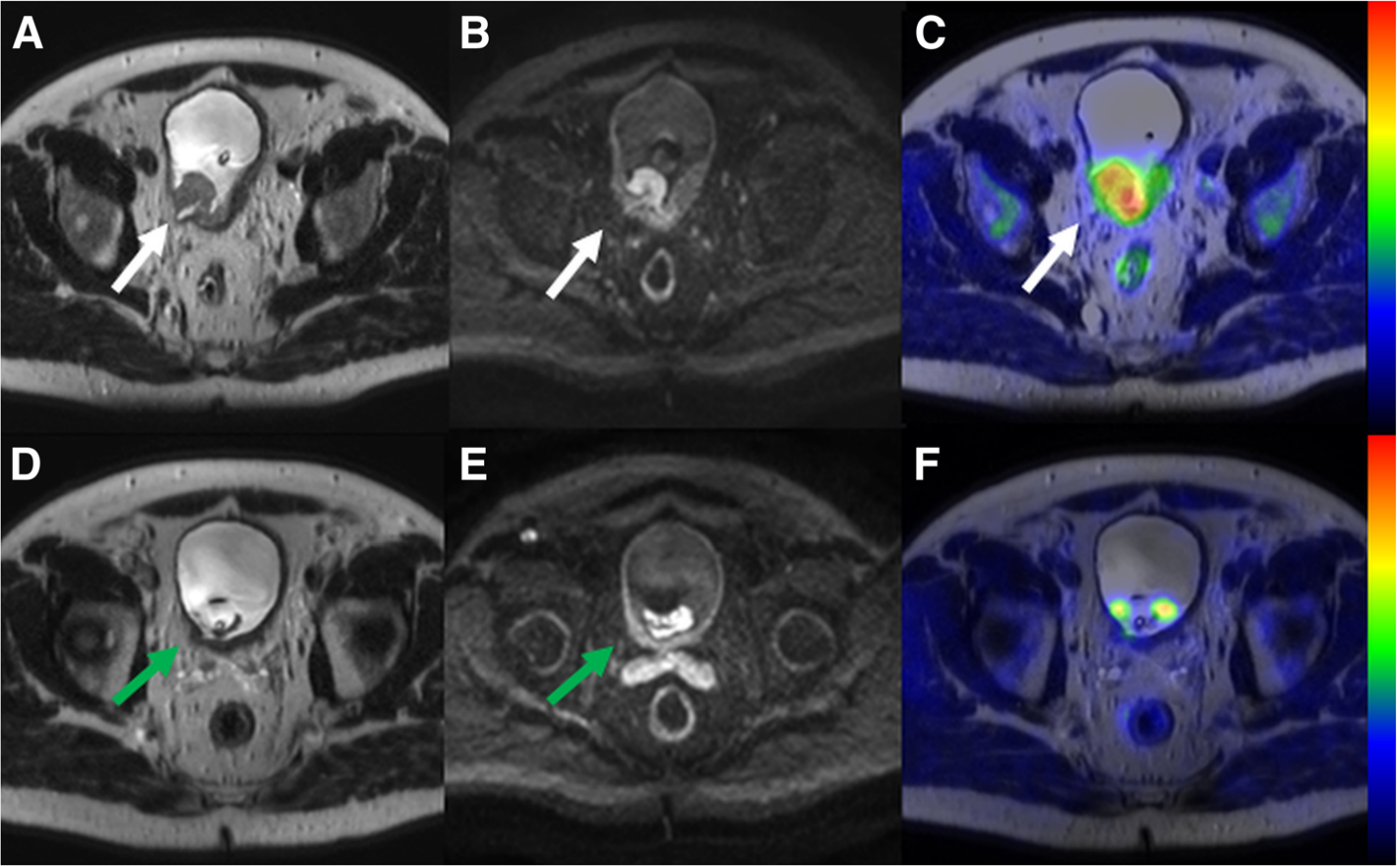
Comparison of local bladder cancer staging (T-stage) on pre-transurethral resection (**a**, **b**, **c**) and post-chemotherapy ^11^C-acetate PET/MRI (**d**, **e**, **f**) in a 63-year-old male patient (number 12 in primary imaging). A heterogenous lobulated mass on the right side of mid-line **with** extension (white arrow) to perivesical fat seen on T2-weighted image (**a**), an area of increased diffusion signal restriction (white arrow) beyond the bladder wall (**b** - b value 800 s/mm^2^ trace diffusion weighted image), an associated right sided hydroureter, and increased ^11^C-acetate uptake (**c** - PET fused with T2-weighted image, SUV is scaled from 0.0 to 3.5) suggestive of T3 stage. On post-chemotherapy ^11^C-acetate PET/MRI, residual abnormal wall thickening (green arrow) on T2-weighted image (**d**) and diffusion signal restriction extending to perivesical fat (**e** - b value 800 s/mm^2^ trace diffusion weighted image) was presented suggestive of T3 stage. The final cystectomy specimen revealed stage T2, thus the findings of ^11^C-acetate PET/MRI were considered as true positive for muscle invasion

The sensitivity, specificity, accuracy and AUC (95% confidence interval) values of ^11^C-acetate PET/MRI for the detection of MIBC (primary BT staging) were 1.00, 0.69 and 0.73, and 0.85 (0.55–1.00), respectively (Figs. 3 and 4). The individual differences for all 15 patients between ^11^C-acetate PET/MRI and TUR-BT are presented in Table 2.

**Table 2:** ^11^C-acetate PET/MRI primary staging

Patient no	PET-MRI	TUR-BT pathology
1	T1	Tis
2	T1	Ta
3	T1	T1
4	T2	T1
5	T2	T1
6	T3	T2
7	T1	T1
8	T1	Ta
9	T1	Ta
10	T1	Ta
11	T1	T1
12	T3	T2
13	T3	T1
14	T2	Ta
15	T1	T1

*PET/MRI* positron emission tomography – magnetic resonance imaging, *TUR-BT* transurethral resection of bladder tumor. T-stage according to the TNM classification

### Therapy response evaluation

**Fig. 5:**
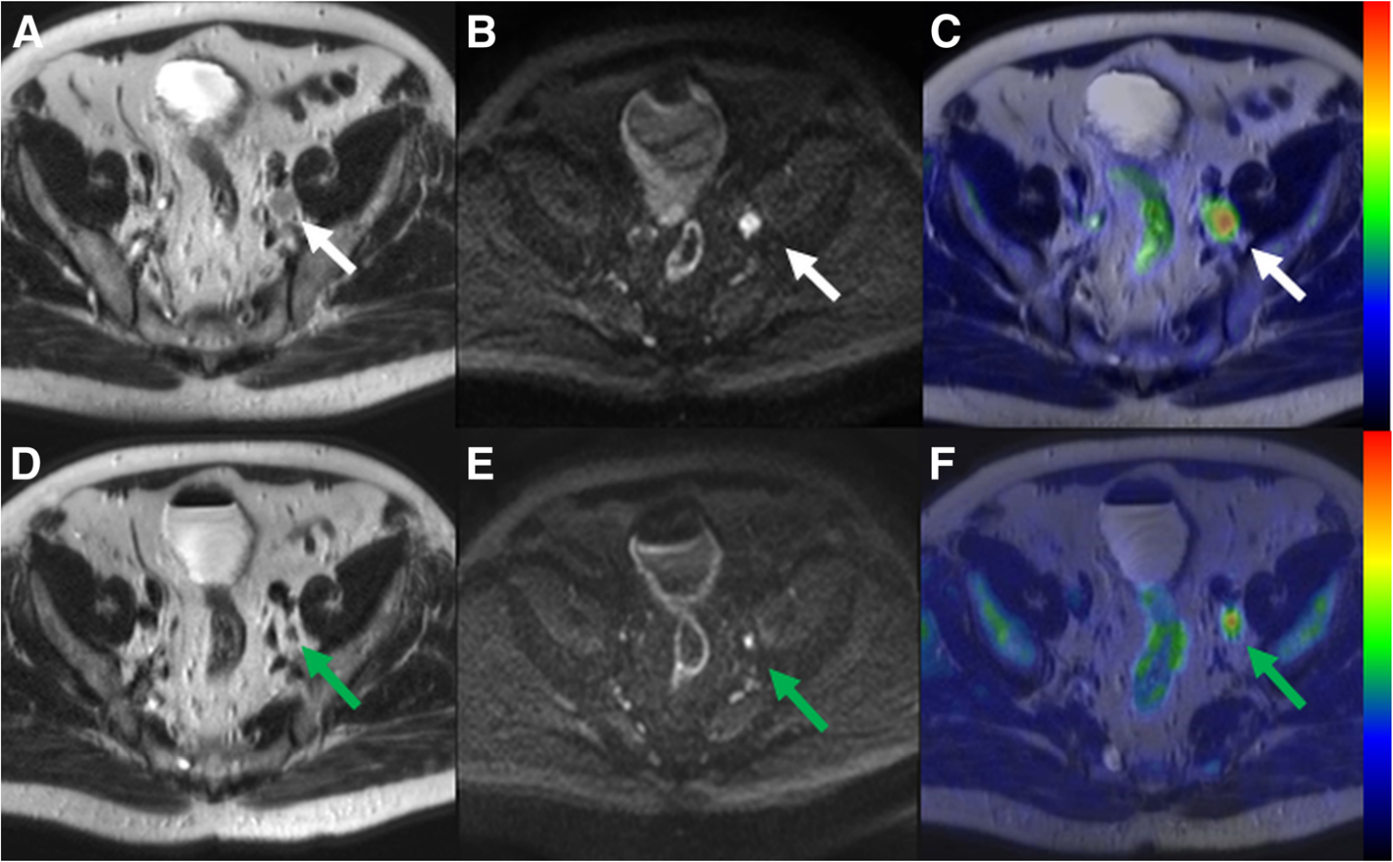
Comparison of lymph node staging (N-stage) on pre-transurethral resection (**a**, **b**, **c**) and post-chemotherapy ^11^C-acetate PET/MRI (**d**, **e**, **f**) in patient number 12, the same patient as in Fig. 4. 17 mm right obturator lymph node (**a** - white arrow on T2-weighted image) demonstrated increased diffusion signal restriction (**b** - b value 800 s/mm^2^ trace diffusion weighted image) and increased ^11^C-acetate uptake (**c** - PET fused with T2-weighted image, SUV is scaled from 0.0 to 3.5) suggestive of lymph node metastasis. On post-chemotherapy ^11^C-acetate PET/MRI, lymph node decreased in size and measured 4 mm (**d** - green arrow on T2-weighted image) with increased diffusion signal (**e** - b value 800 s/mm^2^ trace diffusion weighted image) and increased ^11^C-acetate uptake (**f** - PET fused with T2-weighted image, SUV is scaled from 0.0 to 3.0). SUVmax values of the lymph node on the pre-transurethral resection (**c**) and post-chemotherapy ^11^C-acetate PET/MRI (**f**) were 3.4 and 2.8, respectively. The lymph node was confirmed to be lymph node metastasis measuring 3 mm on extended pelvic lymph node dissection, thus the findings of ^11^C-acetate PET/MRI were considered as true positive

**Fig. 6:**
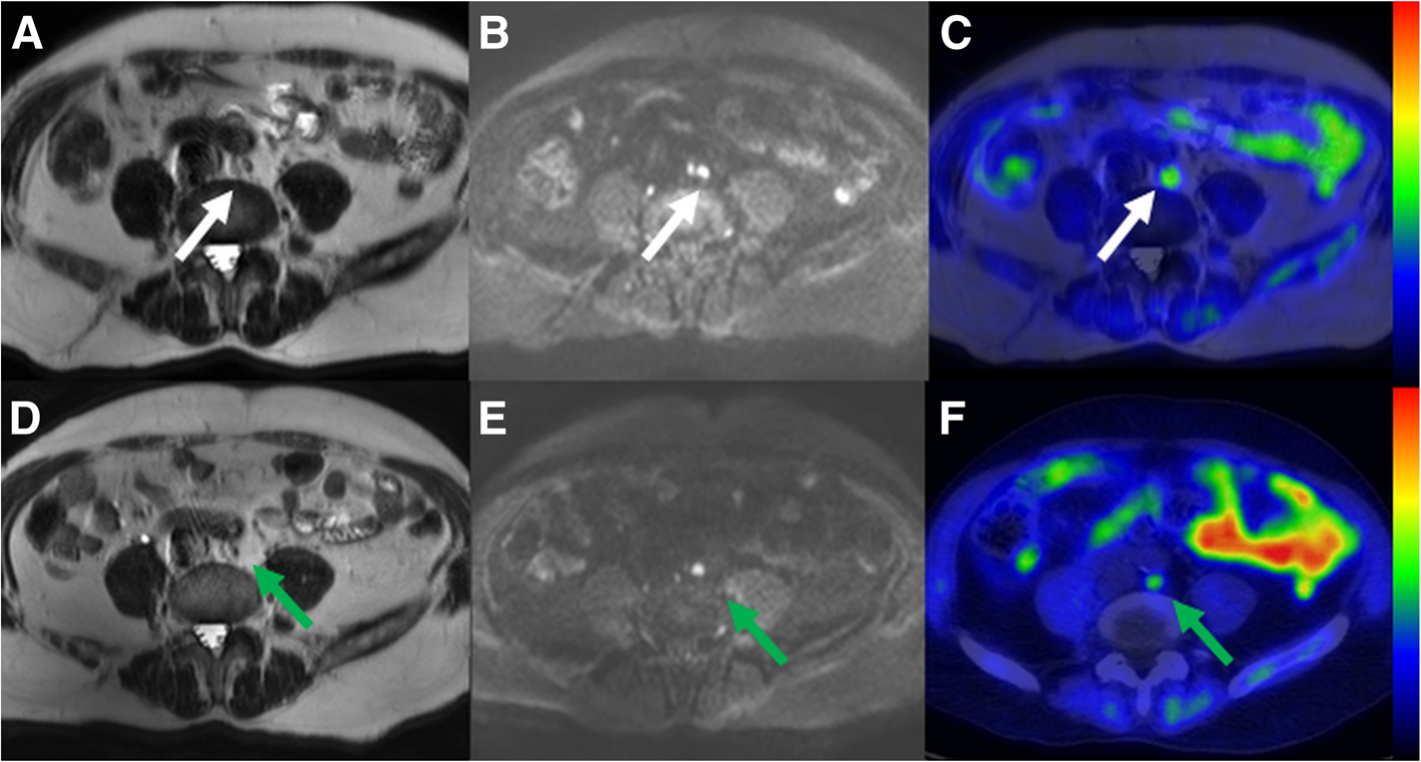
Comparison of lymph node staging (N-stage) on pre-transurethral resection (**a**, **b**, **c**) and post-chemotherapy ^11^C-acetate PET/MRI (**d**, **e**, **f**) in a 66-year-old male (patient number 6 in primary imaging). 8 mm retroaortic lymph node (**d** - white arrow on T2-weighted image) demonstrated increased diffusion signal restriction (**b** - b value 800 s/mm^2^ trace diffusion weighted image), and increased ^11^C-acetate uptake (**c** - PET fused with T2-weighted image, SUV is scaled from 0.0 to 2.8) suggestive of lymph node metastasis. On post-chemotherapy ^11^C-acetate PET/MRI, lymph did not decrease in size and measured 7 mm (**d** - green arrow on T2-weighted image) with increased diffusion signal (**e** - b value 800 s/mm^2^ trace diffusion weighted image) and increased ^11^C-acetate uptake (**f** - PET fused with T2-weighted image, SUV is scaled from 0.0 to 2.8). SUVmax values of the lymph node on the pre-transurethral resection (**c**) and post-chemotherapy ^11^C-acetate PET/MRI (**f**) was 1.7, 1.3, respectively. No lymph node metastases were found on extended pelvic lymph node dissection, thus the findings of ^11^C-acetate PET/MRI were considered as false positive

All five patients with MIBC underwent NAC and had RC and EPLND. NAC was generally well tolerated and all 5 patients received the planned amount of cycles but dose adjustment of anticancer drugs was necessary for 2 patients before surgery. Comparison of ^11^C-acetate PET/MRI and histopathological evaluation on patient level are presented in Table 3. Compared to histopathology ^11^C-acetate PET/MRI correctly staged three patients (Figs. 4 and 5), overstaged one (Fig. 6) and understaged one patient. True negative findings in BT were reported in two patients and true positive in one patient, respectively. Details are presented in the Supporting Material.

**Table 3:** Treatment response evaluation to neoadjuvant chemotherapy using ^11^C-acetate PET/MRI. Patients 1 and 2 participated in both phases of the trial, underwent PET/MRI before any intervention and secondary PET/MRI was performed after completion of NAC. Patients 3–5 were enrolled after TUR-BT and underwent PET/MRI after completion of NAC. Imaging after NAC prior RC was compared to RC pathology

Patient	PET/MRI		RC	
	Primary tumour	LNM +/−	Primary tumour	LN n tot / n+
1	T0	+	T0	27 / 0
2	T4	+	T2	42 / 3
3^a^	T0	–	T3	27 / 9
4	T0	–	T0	43 / 0
5	T3	–	T2	36 / 0
				175 / 12

*PET/MRI* positron emission tomography – magnetic resonance imaging, *RC* radical cystectomy, *LNM* lymph node metastasis (+) = suspicion of presence; (−) = suspicion of absence

^a^Patient had bilateral hip prostheses which distorted the image quality

### Lymph node metastases detection

The median number of evaluated nodes per patient was 35 (range 27–43). Of a total 175 LNs removed, 12 (7%) harboured metastases. On patient level, two of the five patients had nodal metastatic disease (Table 4, Fig. 5). All metastatic LNs were found around the iliac arteries and none were identified outside the pelvis (Fig. 6). The sensitivity, specificity and accuracy of ^11^C-acetate PET/MRI for the detection of LN metastases on the predetermined 10 nodal areas were 0.2, 0.96, 0.88, respectively, and AUC (95% confidence interval) value was 0.58. The corresponding values on patient level were 0.5, 0.67, 0.6, and 0.58 respectively (Table 4).

**Table 4:** ^11^C-acetate PET/MRI lymph node evaluation

Area of interest	Sensitivity	Specificity	Accuracy	AUC
LN areas (*n* = 50)	0.20	0.96	0.88	0.58
On patient level^a^ (*n* = 5)	0.50	0.67	0.60	0.58

^a^evaluation of LN positivity. Only 2 patients had LN metastases and the other had hip prostheses

## Discussion

This prospective registered clinical trial is the first study to demonstrate feasibility of ^11^C-acetate PET/MRI in BC staging and its potential to assist in monitoring response to NAC. Compared to CT, MRI as the anatomic modality may be favoured since CT is poor for evaluation of muscle invasion. Similar to prostate cancer [24], ^11^C- acetate PET/MRI does not seem to offer a satisfactory solution for the detection of metastatic LNs in pelvis due to limited accuracy.

^11^C-acetate PET/MRI demonstrated accuracy of 0.73 in primary tumor staging, detection of MIBC. In previous studies higher accuracy of MRI alone has been reported [25]. Green et al. [25] noted, however, that differentiation between T1 high grade tumor and T2 tumor is challenging even with known histopathology and TUR-BT stage. Two studies of PET/MRI after TUR-BT or with proven history of BC using 2-deoxy-2-[^18^F]fluoro-D-glucose (^18^F-FDG) report high activity in urinary system and in inflammatory tissue [26, 27]. This is clearly detrimental for evaluation of BT because of suboptimal target-to-background ratio. In the current trial, treatment naive patients with suspicion of muscle invasion in cystoscopy were imaged minimizing the impact of inflammatory reaction post TUR-BT. ^11^C-acetate did not miss any MIBC cases and we hypothesise that ^11^C- acetate could outperform ^18^F-FDG since accuracy of ^18^F-FDG is limited by urine extraction. Although overstaging affected accuracy in the current trial, it is less significant as clinical challenge than understaging, which is a common cause of treatment delay, meaning execution of RC and ePLND.

Only three studies, all published in 2012, reported evaluation of ^11^C-acetate PET/CT in bladder cancer. Vargas et al. reported on 16 patients and compared MRI, CT and ^11^C-acetate PET/CT before cystectomy and PLND. They concluded that while all three modalities had similar accuracy PET/CT carried a risk of understaging [28]. Orevi et al. found in a study of 13 patients that ^11^C-acetate PET/CT was positive in 10 LNs, of which five were malignant giving a specificity of 50% [13]. Finally, Schröder et al. demonstrated that ^11^C-acetate PET/CT showed specificity of 50% and sensitivity of 80% for the detection of LN metastases. They also noted that intravesical instillation therapy with BCG yielded falsely high positive findings in the resected tumor bed of bladder wall as well as in LNs. [15]. Similar problems were seen by Vargas et al. [28]. In comparison to all these three PET/CT studies [13, 15, 28] in the current trial, specificity of 50% on patient level for detection of regional metastases was found. If one patient with hip prostheses was discarded, sensitivity would have been 100%. It is tempting to assume that ^11^C-acetate PET/MRI is comparable or better than PET/CT for primary tumor evaluation. However, there are no studies directly comparing PET/CT to PET/MRI in bladder cancer staging and, therefore, the issue remains a matter of further research.

Our registered prospective trial is the third ^11^C -acetate study evaluating LNs in BC. However, ^11^C -acetate and PET-MRI have not been used as a surrogate modality before [13, 28, 29]. A pilot study demonstrating the utility of ^18^F-FDG PET/MRI in BC staging has already been conducted [26]. The results seem to favour PET/MRI in LN evaluation compared to MRI alone, but the number of study subjects was low and needs to be verified in larger studies. Orevi et al. demonstrated that ^11^C-choline and acetate were comparable in evaluation of lymph node metastases [13], and recent meta-analytic study [29] reports for both tracers low sensitivity and moderate specificity while heterogeneity of publications limits further conclusions. Our sensitivity of 20% in LN staging is in line with the meta-analysis and we could conclude that the optimal tracer for evaluating urinary tract and pelvic LNs remains to be found.

Studies comparing PET/CT vs. PET/MRI for the detection of lymph nodes are lacking. ^18^F-FDG PET is widely available and has been studied in multiple studies enrolling BC patients, but prior studies were mainly performed with PET/CT and some compared to CT alone [30–32]. Overall, the results are conflicting. Swinnen et al. found no benefit from adding PET to CT alone [30]. ^18^F-FDG is highly active in urinary system and forced urinary protocols and delayed execution of PET imaging have been shown to improve performance of ^18^F-FDG PET/CT [33]. Using these methods, excessive hydration and use of diuretics can also be considered an extra burden for the patient. Although the published evidence does not support routine use of PET-imaging for BC staging, our results indicate that ^11^C-acetate is a viable tracer option in BC staging.

Our study has several limitations. First, the number of patients especially evaluated for NAC response was low. Furthermore, patient no. 3 in NAC treatment response group had bilateral hip prostheses, which caused B_0_ field distortions and the image quality was not optimal. We hypothesize that the patient’s largest metastatic 2.0 cm LN would not have been missed if B_0_ field distortions were fully compensated. Although excellent therapy responses were found (Figs. 4 and 5) the low number of patients precludes definite conclusion about the value of ^11^C -acetate PET/MRI in setting of therapy response evaluation. In contrast, compared to previous studies a higher number of treatment (*n* = 15) naive patients underwent baseline imaging, and allowed us to evaluate performance of ^11^C -acetate PET/MRI without contribution of inflammation to findings. To decrease heterogeneity in future studies, BCG or chemotherapy ideally should not be given before initial PET-MRI or PET-CT. In the current trial T1-weighted imaging and dynamic contract enhanced MRI were not performed. Before conducting this trial, we have performed very careful optimization of MRI acquisition protocol, done multiple iterations of the acquisition protocol, and carefully optimized the acquisition protocol with special attention on DWI. As can be seen in Figs. 3, 4, 5 and 6, DWI was the “workhorse “for TNM staging of BC. In order to increase openness of our trial, promote #opensource research (#OpenSourceTrial), we share our optimized MRI acquisition protocol in supporting material.

## Conclusion

In conclusion, we found a moderate accuracy for staging of primary BC using ^11^C-acetate PET/MRI in this pilot prospective registered clinical trial. In contrast, only a limited sensitivity for detection of metastatic lymph nodes and response to neoadjuvant chemotherapy was found. Our findings do not advocate for routine use of ^11^C-acetate PET/MRI in staging of BC but consideration of its potential role in future organ preservation trials with combined use of imaging and other markers, such as molecular information, is warranted.

## Abbreviations

AUC: Area under curve
BC: Bladder cancer
BCG: Bacillus-Calmette-Guerin bladder instillation therapy
CT: Computed tomography
DWI: Diffusion weighted imaging
FDG: 2-deoxy-2-[^18^F]fluoro-D-glucose
FOV: Field of view
LM-OSEM: Maximum-likelihood ordered subsets expectation maximum
MIBC: Muscle invasive bladder cancer
MRAC: MR-based attenuation correction
MRI: Magnetic resonance imaging
NAC: Neoadjuvant chemotherapy
PET/CT: Postiron emission tomography – Computed tomography
PET/MRI: Positron emission tomography – Magnetic resonance imaging
PLND: Pelvic lymph node dissection
RC: Radical cystectomy
SUV: Standardized uptake value
TE: Echo Time
Tis: Tumor in situ
TNM: Tumor – node – metastases Classification for malignant tumors
TR: Repetition Time
TUR-BT: Transurethral resection of bladder tumor
USPIO: Ultra small paramagnetic particles of iron oxide

## References

[1] https://seer.cancer.gov/statfacts/html/urinb.html (site visited 6.12.2017).

[2] https://ec.europa.eu/jrc/en/publication/epidemiology-bladder-cancer-europe (site visited 30.5.2018).

[3] Brierley JD, Gospodarowicz MK, Wittekind C (2009). TNM Classification of Malignant Tumours, 7th Edition.

[4] Anastasiadis A, de Reijke TM (2012). Best practice in the treatment of nonmuscle invasive bladder cancer. Ther Adv Urol.

[5] Alfred Witjes J, Lebret T, Compérat EM, Cowan NC, De Santis M, Bruins HM, Hernández V, Espinós EL, Dunn J, Rouanne M, Neuzillet Y, Veskimäe E, van der Heijden AG, Gakis G, Ribal MJ. Updated 2016 EAU guidelines on muscle-invasive and metastatic bladder Cancer. Eur Urol. 2016; 10.1016/j.eururo.2016.06.020 27375033

[6] Advanced Bladder Cancer Overview Collaboration (2005). Neoadjuvant chemotherapy for invasive bladder cancer. Cochrane Database Syst Rev.

[7] Shariat S (2007). Discrepancy between Clinical and Pathologic Stage: Impact on Prognosis after Radical Cystectomy. Eur Urol.

[8] Turker P, Bostrom PJ, Wroclawski ML, van Rhijn B, Kortekangas H, Kuk C, Mirtti T, Fleshner NE, Jewett MA, Finelli A, Kwast TV, Evans A, Sweet J, Laato M, Zlotta AR (2012). Upstaging of urothelial cancer at the time of radical cystectomy: factors associated with upstaging and its effect on outcome. BJU Int.

[9] Lin WC, Chen JH (2015). Pitfalls and limitations of diffusion-weighted magnetic resonance imaging in the diagnosis of urinary bladder Cancer. Transl Oncol.

[10] Birkhäuser FD, Studer UE, Froehlich JM, Triantafyllou M, Bains LJ, Petralia G, Vermathen P, Fleischmann A, Thoeny HC (2013). Combined ultrasmall superparamagnetic particles of iron oxide-enhanced and diffusion-weighted magnetic resonance imaging facilitates detection of metastases in normal-sized pelvic lymph nodes of patients with bladder and prostate cancer. Eur Urol.

[11] Triantafyllou M, Studer UE, Birkhäuser FD, Fleischmann A, Bains LJ, Petralia G, Christe A, Froehlich JM, Thoeny HC (2013). Ultrasmall superparamagnetic particles of iron oxide allow for the detection of metastases in normal sized pelvic lymph nodes of patients with bladder and/or prostate cancer. J Cancer.

[12] Goodfellow H, Viney Z, Hughes P, Rankin S, Rottenberg G, Hughes S, Evison F, Dasgupta P, O’Brien T, Khan MS (2014). Role of fluorodeoxyglucose positron emission tomography (FDG PET)-computed tomography (CT) in the staging of bladder cancer. BJU Int.

[13] Orevi M, Klein M, Mishani E, Chisin R, Freedman N, Gofrit ON (2012). 11C-acetate PET/CT in bladder urothelial carcinoma: intraindividual comparison with 11C-choline. Clin Nucl Med.

[14] Maurer T, Horn T, Souvatzoglou M, Eiber M, Beer AJ, Heck MM, Haller B, Gschwend JE, Schwaiger M, Treiber U, Krause BJ (2014). Prognostic value of 11C-choline PET/CT and CT for predicting survival of bladder cancer patients treated with radical cystectomy. Urol Int.

[15] Schöder H, Ong SC, Reuter VE, Cai S, Burnazi E, Dalbagni G, Larson SM, Bochner BH (2012). Initial results with (11)C-acetate positron emission tomography/computed tomography (PET/CT) in the staging of urinary bladder cancer. Mol Imaging Biol.

[16] Rosenkrantz AB, Friedman K, Chandarana H, Melsaether A, Moy L, Ding YS, Jhaveri K, Beltran L, Jain R (2015). Current status of hybrid PET/MRI in oncologic imaging. AJR Am J Roentgenol.

[17] Zaidi H, Ojha N, Morich M (2011). Design and performance evaluation of a whole-body ingenuity TF PET/MRI system. Phys Med Biol.

[18] Pruessmann KP, Weiger M, Scheidegger MB, Boesiger P (1999). SENSE: sensitivity encoding for fast MRI. Magn Reson Med.

[19] Jambor I, Pesola M, Merisaari H, Taimen P, Boström PJ, Liimatainen T, Aronen HJ (2016). Relaxation along fictitious field, diffusion-weighted imaging, and T2 mapping of prostate cancer: prediction of cancer aggressiveness. Magn Reson Med.

[20] Gandhi N, Krishna S, Booth CM, Breau RH, Flood TA, Morgan SC, Schieda N, Salameh JP, McGrath TA, McInnes M. Diagnostic accuracy of MRI for tumor staging of bladder cancer: systematic review and meta-analysis. BJU Int. 2018; 10.1111/bju.14366. [Epub ahead of print] 10.1111/bju.14366 29727910

[21] Babjuk M, Böhle A, Burger CO, Cohen D, Compérat EM, Hernández V, Kaasinen E, Palou J, Rouprêt M, van Rhijn BW, Shariat SF, Soukup V, Sylvester RJ, Zigeuner R. EAU Guidelines on Non-Muscle-invasive Urothelial Carcinoma of the Bladder: Update 2016, Eur Urol. 2016. 10.1016/j.eururo.2016.05.041 27324428

[22] Roth B, Wissmeyer MP, Zehnder P, Birkhäuser FD, Thalmann GN, Krause TM, Studer UE (2010). A new multimodality technique accurately maps the primary lymphatic landing sites of the bladder. Eur Urol.

[23] DeGroot MH, Schervish MJ (2011). Kolmogorov–Smirnov tests. Prob- ability and statistics.

[24] Jambor I, Borra R, Kemppainen J, Lepomäki V, Parkkola R, Dean K, Alanen K, Arponen E, Nurmi M, Aronen HJ, Minn H (2012). Improved detection of localized prostate cancer using co-registered MRI and 11C-acetate PET/CT. Eur J Radiol.

[25] Green DA, Durand M, Gumpeni N, Rink M, Cha EK, Karakiewicz PI, Scherr DS, Shariat SF (2012). Role of magnetic resonance imaging in bladder cancer: current status and emerging techniques. BJU Int.

[26] Rosenkrantz AB, Balar AV, Huang WC, Jackson K, Friedman KP (2015). Comparison of Coregistration accuracy of pelvic structures between sequential and simultaneous imaging during hybrid PET/MRI in patients with bladder Cancer. Clin Nucl Med.

[27] Rosenkrantz AB, Friedman KP, Ponzo F, Raad RA, Jackson K, Huang WC, Balar AV (2017). Prospective pilot study to evaluate the incremental value of PET information in patients with bladder Cancer undergoing 18F-FDG simultaneous PET/MRI. Clin Nucl Med.

[28] Vargas HA, Akin O, Schoder H, et al. Prospective evaluation of MRI, (^11^)C-acetate PET/CT and contrast-enhanced CT for staging of bladder cancer. Eur J Radiol. 2012; 10.1016/j.ejrad.2012.06.010 22858427

[29] Kim SJ, Koo PJ, Pak K, Kim IJ, Kim K (2018). Diagnostic accuracy of C-11 choline and C-11 acetate for lymph node staging in patients with bladder cancer: a systematic review and meta-analysis. World J Urol.

[30] Swinnen G, Maes A, Pottel H, Vanneste A, Billiet I, Lesage K (2010). FDG-PET/CT for the preoperative lymph node staging of invasive bladder cancer. Eur Urol.

[31] Lodde M, Lacombe L, Friede J, Morin F, Saourine A, Fradet Y (2010). Evaluation of fluorodeoxyglucose positron-emission tomography with computed tomography for staging of urothelial carcinoma. BJU Int.

[32] Kibel AS, Dehdashti F, Katz MD, Klim AP, Grubb RL, Humphrey PA (2009). Prospective study of ^18^F-fluorodeoxyglucose positron emission tomography/computed tomography for staging of muscle-invasive bladder carcinoma. J Clin Oncol.

[33] Harkirat S, Anand S, Jacob M (2010). Forced diuresis and dual-phase F-fluorodeoxyglucose-PET/CT scan for restaging of urinary bladder cancers. Indian J Radiol Imaging.

